# Interdependence between network dynamics and connectivity in dissociated cortical cultures: a theoretical and experimental approach

**DOI:** 10.1186/1471-2202-12-S1-P214

**Published:** 2011-07-18

**Authors:** Paolo Massobrio, Valentina Pasquale, Matteo Garofalo, Sergio Martinoia

**Affiliations:** 1 Department of Biophysical and Electronic Engineering (DIBE), University of Genova, Genova, Italy; 2 Department of Neuroscience and Brain Technologies, Italian Institute of Technology (IIT), Genova, Italy

## 

In this work we aimed at investigating the interdependence between connectivity and dynamics in large-scale cortical networks cultured *in vitro* onto planar Micro-Electrode Arrays (MEAs). In this experimental model, neurons are free of predefined constraints and thus able to re-create networks that exhibit complex and highly variable spatio-temporal patterns of activity composed of synchronized bursts, mixed with random spikes. Starting from this experimental evidence, here we address the questions: does a particular network architecture promote such dynamics? Is it possible to predict the activity of a neuronal network on the basis of its connectivity map?

We determined the dynamic state of the network according to the statistical distribution of *neuronal avalanches* (namely critical, sub-critical or super-critical) [1,2]. Due to the difficulties of determining the network topology of our cultures from a limited number of recording sites (60 microelectrodes), we took advantage of a computational model consisting of a neuronal network made up of 1024 Izhikevich neurons [3]. Network topologies were designed following the canonical architectures scale-free, random, and small-world [4]. Within this approach, the network is dealt as a graph, where each neuron corresponds to a node, and each synaptically connections to an edge. We simulated the spontaneous activity of such neuronal networks, by sweeping the most common parameters used to characterize these graphs, such as clustering coefficient, connection density, etc. [5]. The main finding which emerges is that although all the network configurations determine a mix of spiking, and bursting activity, the scale-free and partially small-world architectures display a critical behavior, and thus self-organization could be influenced by the network architecture.

## Conclusions

We used a combined approach involving models and experiments to get an insight into the interplay of topology and dynamics in cortical networks cultured *in vitro*. Our results showed that different topologies of connectivity may determine different dynamic states and suggest that a scale-free architecture may account for the variability observed in the experimental data by varying the number of hubs.
